# The Prevalence and Drug Susceptibility of *Candida* Species and an Analysis of Risk Factors for Oral Candidiasis—A Retrospective Study

**DOI:** 10.3390/antibiotics14090876

**Published:** 2025-08-30

**Authors:** Marcin Tkaczyk, Anna Kuśka-Kielbratowska, Jakub Fiegler-Rudol, Wojciech Niemczyk, Anna Mertas, Dariusz Skaba, Rafał Wiench

**Affiliations:** 1 Department of Periodontal Diseases and Oral Mucosa Diseases, Faculty of Medical Sciences in Zabrze, Medical University of Silesia, 40-055 Katowice, Poland; mtkaczyk@sum.edu.pl (M.T.); anna.kuska-kielbratowska@sum.edu.pl (A.K.-K.); s88998@365.sum.edu.pl (J.F.-R.); rwiench@sum.edu.pl (R.W.); 2 Department of Microbiology and Immunology, Faculty of Medical Sciences in Zabrze, Medical University of Silesia in Katowice, Jordana 19 Str., 41-808 Zabrze, Poland; amertas@sum.edu.pl

**Keywords:** *Candida albicans*, non-albicans *Candida*, denture stomatitis, drug resistance, antifungal susceptibility, species distribution, fungal prevalence

## Abstract

Background: Oral candidiasis is a prevalent opportunistic infection, predominantly caused by *Candida albicans* (*CA*), though non-albicans *Candida* (NAC) species are increasing worldwide. This study aimed to characterize the prevalence of *Candida* species, evaluate antifungal susceptibility, and identify predisposing risk factors in patients with oral mucosal candidiasis. Methods: A retrospective review of 1286 electronic patient medical records (788 women, 498 men) from 2018 to 2022 was conducted at the Department of Periodontal and Oral Mucosa Diseases, Medical University of Silesia. Swabs from the oral cavity were processed to identify *Candida* strains by mass spectrometry, followed by drug susceptibility testing for amphotericin B, nystatin, flucytosine, econazole, ketoconazole, miconazole, and fluconazole. Relevant local and systemic predisposing factors were recorded and analyzed statistically. Results: Among 958 patients with positive fungal cultures, *CA* accounted for 66.79% of isolates, while NAC constituted 33.21%. Multi-strain infections were detected in 8.46% of patients. *CA* showed lower resistance (<10%) to amphotericin B, nystatin, and flucytosine, but up to 30% resistance to azoles. NAC strains demonstrated elevated resistance rates (>40% for most azoles), with *C. krusei* exhibiting the highest resistance to the previously mentioned antifungal agents. Key risk factors included wearing removable dentures (*p* = 0.042) and uncontrolled diabetes mellitus (*p* = 0.0431). Additional factors, including poor oral hygiene, reduced salivary flow, and immunosuppressive conditions, further increased infection risk. Patients presenting with multiple risk factors were more likely to have multi-strain infections and more severe disease courses. Conclusions: This retrospective analysis highlights the growing prevalence of NAC, rising antifungal resistance (particularly to azoles), and the importance of identifying risk factors, especially denture use and poor glycemic control. Enhanced preventive strategies, robust diagnostic approaches, and optimized antifungal regimens are essential to address this evolving clinical challenge.

## 1. Introduction

*Candidiasis* is among the most common infectious diseases of the oral mucosa, and is primarily caused by yeast-like fungi of the genus *Candida* [1], with *Candida albicans* (*CA*) being the most frequent species (47–84% of oral infections [2]). However, epidemiological data reveal a rising incidence of NAC, including *C. glabrata*, *C. tropicalis*, *C. pseudotropicalis*, *C. parapsilosis*, *C. dubliniensis*, *C. guilliermondii*, *C. krusei*, *C. kefyr*, *C. lusitaniae*, and *C. stellatoidea* [3,4,5,6,7,8], driven by prolonged antifungal exposure, improved diagnostics, climate change, and pandemics. NAC pathogens pose significant treatment challenges owing to resistance mechanisms and immune evasion. Environmental pressures have also led to the emergence of rare species such as *C. palmioleophila*, *C. nivariensis*, *C. auris*, *C. spencermartinsiae*, *C. quercitrusa*, *C. pseudohaemulonii*, *C. duobushaemulonii*, *C. sphaerica*, *C. rugosa*, *C. haemulonii*, *C. bracarensis*, and *C. utilis* [9,10,11,12]. Recent years have seen a global surge in *C. auris*, first identified in 2009 in Japan, and now reported in East Asia, Asia Minor, South Africa, the United States, South America, and the UK. This multidrug-resistant fungus causes outbreaks of invasive infections in patients receiving medical care (including those recovering from SARS-CoV-2), oncology patients, and even immunocompetent individuals. It poses a major global threat due to its ease of transmission, resistance to antifungals and disinfectants, numerous virulence factors, and a mortality rate of up to 72% [13,14]. Another challenging strain, *C. palmioleophila*, has been linked to severe endogenous fungal intraocular inflammation [11]. Additionally, *Candida* spp. may contribute to oral epithelial carcinogenesis, particularly squamous cell carcinoma, by converting nitrite and nitrate into nitrosamines, producing acetaldehyde, and secreting candelysin and esterase that promote metaplasia and further predispose cells to precancerous changes [15,16].

The *Stephanoascus ciferrii* complex, which now encompasses *Stephanoascus ciferrii* (syn. *Trichomonascus ciferrii*), *Candida allociferrii*, and *Candida mucifera*, has emerged as a clinical concern by causing both localized (ear and eye) and occasional severe infections in immunocompromised individuals, and it displays reduced susceptibility to common antifungal agents [17]. Worldwide, fungal infections of the oral mucosa, genitals, skin, and other sites have risen in recent decades [18]. While often regarded as superficial in dentistry, candidemia is a serious concern, ranking among the most common hospital bloodstream infections (BSIs) and the principal form of invasive candidiasis, with mortality reaching 50%. In the United States and Europe, *Candida* spp. accounts for over 90% of fungal BSIs, ranking fourth and seventh among all BSIs, respectively, and third among neonates [19,20]. Through tigmotropism, *CA* and NAC can invade intravascular spaces and spread to organs such as the liver, spleen, kidneys, heart, and brain, leading to life-threatening mycoses [21]. *CA* also forms organized, multi-strain biofilms, often with *Streptococcus gordonii*, *S. salivarius*, *S. oralis*, and NAC species, on mucosal and dental surfaces. These biofilms are inherently less sensitive or resistant to antimicrobials due to morphological alterations of the cell wall, including reduced ergosterol and altered beta-glucan structure [22,23,24,25,26]. Finally, inconsistent antifungal therapy, whether due to insufficient treatment duration or suboptimal dosing, contributes to poor outcomes, often necessitating multiple agents at higher doses and increasing the risk of recurrence [27,28,29,30,31].

The objective of this study was to retrospectively evaluate the prevalence and drug susceptibility of *Candida* strains isolated from patients of the Department of Periodontal and Oral Mucosa Diseases in Zabrze, Silesian Medical University in Katowice, who had been diagnosed with oral mucosa candidiasis between 2018 and 2022. Additionally, the study aimed to analyze the risk factors that predispose its development.

## 2. Results

**Figure 1:**
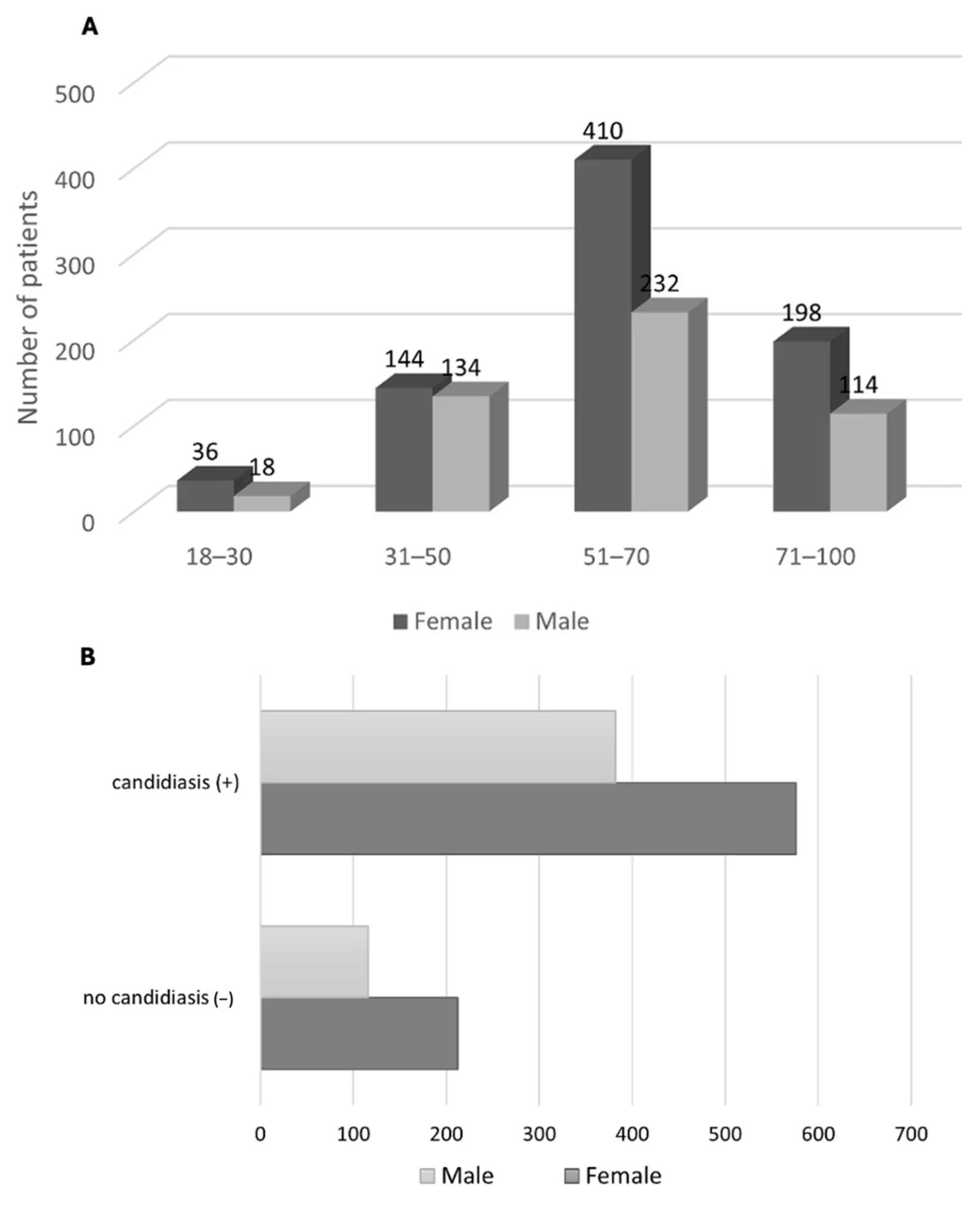
(**A**). Demographic characteristics of patients under evaluation—age group breakdown with gender distinction. (**B**). Characteristics of the incidence of oral mucosal candidiasis in men and women. No statistically significant relationship between oral mucosal candidiasis and gender (*p* = 0.1479).

The medical records of 1286 patients admitted to the Periodontal and Oral Mucosa Clinic of the Medical University of Silesia in Katowice between 2018 and 2022 were analyzed. The preliminary diagnosis of oral candidiasis was made on the basis of clinical symptoms exhibited by the patients. The study group comprised 788 women and 498 men, with an age range of 18 to 100 years for the former and 18 to 90 years for the latter. The patients were divided into four age groups, with 18–30 years comprising 36 women and 18 men, 31–50 years comprising 144 women and 134 men, 51–70 years comprising 410 women and 232 men, and 71–100 years comprising 198 women and 114 men. A summary of the demographic characteristics of the patients is presented (Figure 1A).

A review of personal data by age group and gender distinction revealed that the largest representation among patients with clinical symptoms of candidiasis was observed in the 51–70 age range (642 patients, 410 women, and 232 men), followed by the 71–100 age range (312 patients, 198 women, and 114 men). The 31–50 age range exhibited the lowest representation (278 patients, 144 women, and 134 men), while the 18–30 age range demonstrated the least representation (54 patients, 36 women and 18 men). Among the 1286 patients examined, a positive mycological examination (quantitative interpretation: 1–4) was observed in 958 patients (576 women and 382 men) (Figure 1B).

The retrospective evaluation revealed that mycological examinations yielded a total of 1102 *Candida* strains isolated from 958 patients with a positive test result. Among these, 81 patients (8.46%) were found to have multispecies infections. Table 1 illustrates the distribution of various *Candida* strains.

The highest percentage of identified strains was in *CA* (736 infections, representing 66.79% of the total), followed by *C. glabrata* (108 infections, 9.80%), *C. tropicalis* (80 infections, 7.26%), and other strains. The remaining identified strains included *C. krusei* (49 infections, 4.45%), *C. dubliniensis* (44 infections, 3.99%), *C. kefyr* (41 infections, 3.72%), *C. lusitaniae* (19 infections, 1.72%), and *C. parapsilosis* (16 infections, 1.45%).

The prevalence of NAC infections was found to be 33.21%. Additionally, nine infections (0.82%) were identified, representing rare strains such as *C. stellatoidea*, *C. palmioleophila*, and *C. nivariensis*. A total of 736 strains of *CA* were identified (443 among women and 293 among men), while 377 strains of NAC were identified (221 among women and 145 among men). The study revealed no statistically significant correlation between the strain type and gender (*p* = 0.9515).

The retrospective analysis conducted in our study identified multi-strain infections in 81 patients, with 51 among women and 30 among men; 8.46% of the total number of patients with a positive mycological examination (the most common co-occurrences were *CA* with *C. glabrata*, *CA* with *C. tropicalis*, and *CA* with *C. krusei*, while among NAC it was *C. glabrata* with *C. tropicalis*). Infection with two strains was identified in 37 patients (3.86% of the total number of mycological positive patients), with three strains in 25 patients (2.61% of the total number of mycological positive patients), and with four strains in 19 patients (1.98% of the total number of mycological positive patients). The study demonstrated that there was no statistically significant correlation between multi-strain infection and gender (*p* = 0.5899).

A total of 178 patients exhibited recurrence over five years. Among these patients, 112 were women and 66 were men, representing 18.58% of the total number of patients with a positive mycological examination. The most prevalent site of oral candidiasis was the palate, followed by infection localized on the denture plate, and less frequently on the tongue, cheeks, and corners of the mouth. Other etiologies, including bacterial and viral infections, were identified in 19 patients (1.71%), while candidiasis also manifested in locations outside the oral cavity in 13 patients (1.17%). These included the skin, hand, foot, nails, genitals, and the entire body. The data revealed no statistically significant correlation between recurrence and gender (*p* = 0.3985). The most prevalent form of candidiasis observed in the oral cavity, based on distinctive characteristics, was chronic erythematous candidiasis (presenting with symptoms of prosthetic stomatitis and inflammation of the angles of the lips) (42.92%—552 of the total patients), followed by acute pseudomembranous candidiasis (26.83%—345 patients). A total of 323 patients (25.12%) were diagnosed with keratosis and superinfection with fungi, including lichen planus, leukoplakia, and lupus erythematosus. Hyperplastic candidiasis was identified in 49 patients (3.81%), erythematous acute in 42 patients (3.27%), and pseudomembranous chronic in 36 patients (2.80%). Rhomboid inflammation of the middle part of the tongue was diagnosed in 29 patients (2.26% of the total number of patients).

**Figure 2:**
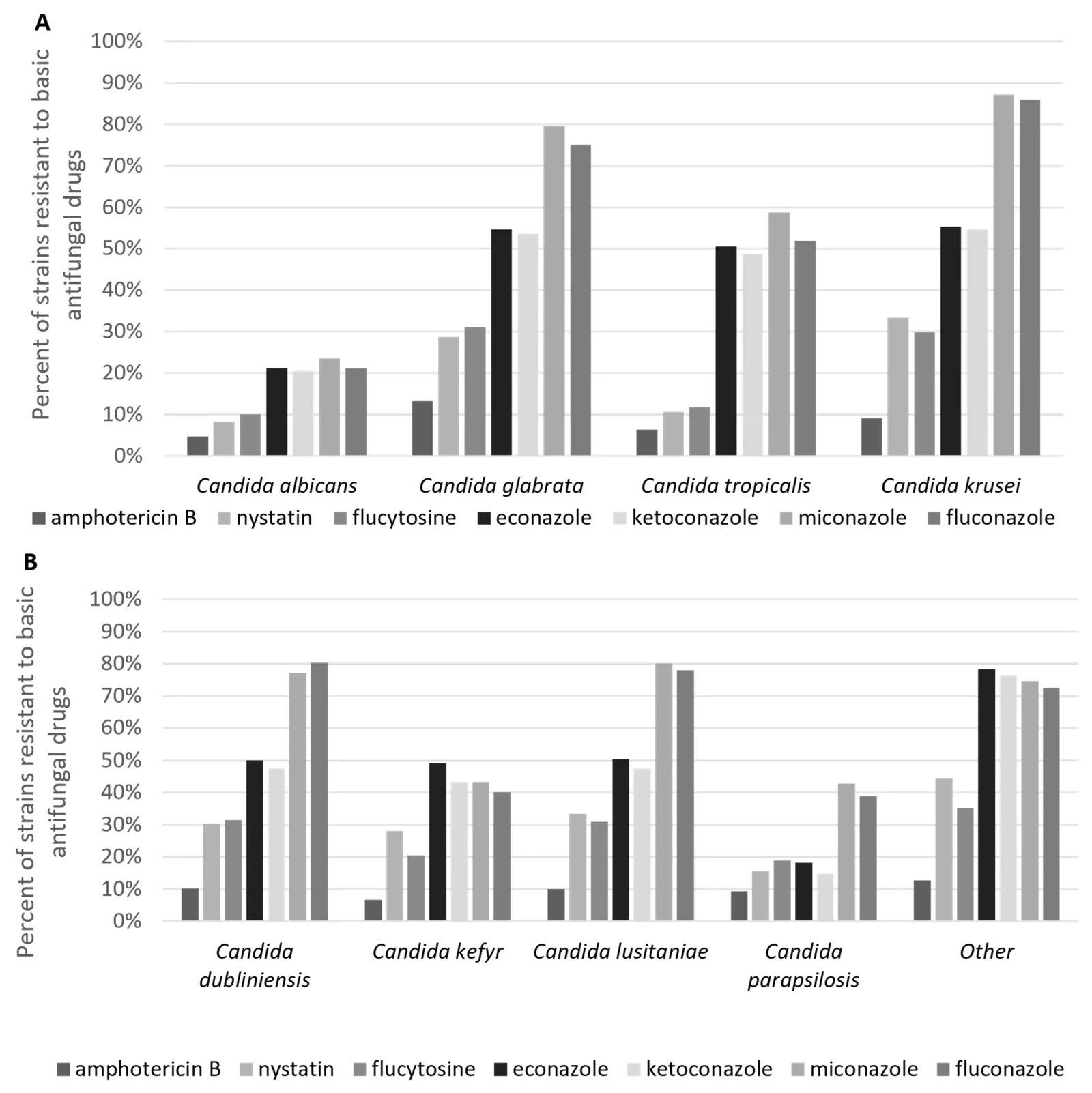
Comparison of resistance of isolated Candida species to basic antifungal drugs. (**A**). Species: *Candida albicans*, *Candida glabrata*, *Candida tropicalis*, and *Candida krusei*. (**B**). Species: *Candida dubliniensis*, *Candida kefyr*, *Candida lusitaniae*, *Candida parapsilosis*, and others.

The data obtained permitted the assessment of drug susceptibility of species of *Candida*, including *CA*, *C. glabrata*, *C. tropicalis*, *C. krusei*, *C. dubliniensis*, *C. kefyr*, *C. lusitaniae*, *C. parapsilosis* and *C. stellatoidea*. The results demonstrated resistance to seven primary antimycotics, including amphotericin B, nystatin, flucytosine, econazole, ketoconazole, miconazole, and fluconazole (Figure 2A,B). Regarding *CA*, as the most identified species, drug resistance of less than 10% to amphotericin B, nystatin, and flucytosine was observed. In contrast, drug resistance to econazole, ketoconazole, miconazole, and fluconazole reached 30%. Most NAC strains (except for *C. parapsilosis*) demonstrated a high level of resistance to azole drugs, with a resistance rate exceeding 40%. *C. krusei* exhibited the highest level of drug resistance. Amphotericin B was identified as the most effective antifungal drug, exhibiting drug resistance below 15% across all strains tested. Nystatin demonstrated interaction with *CA* and exhibited drug resistance in 8.23% of the tested cases, with drug resistance levels below 20%. *C. tropicalis* and *C. parapsilosis* exhibited drug resistance in 30% of cases. The highest levels of drug resistance were observed for miconazole and fluconazole, with over 50% of NAC strains (excluding *C. kefyr* and *C. parapsilosis*) exhibiting resistance.

A review of the data collected revealed local factors that may contribute to an increased risk of oral mucosal candidiasis among the study group (Table 2).

**Figure 3:**
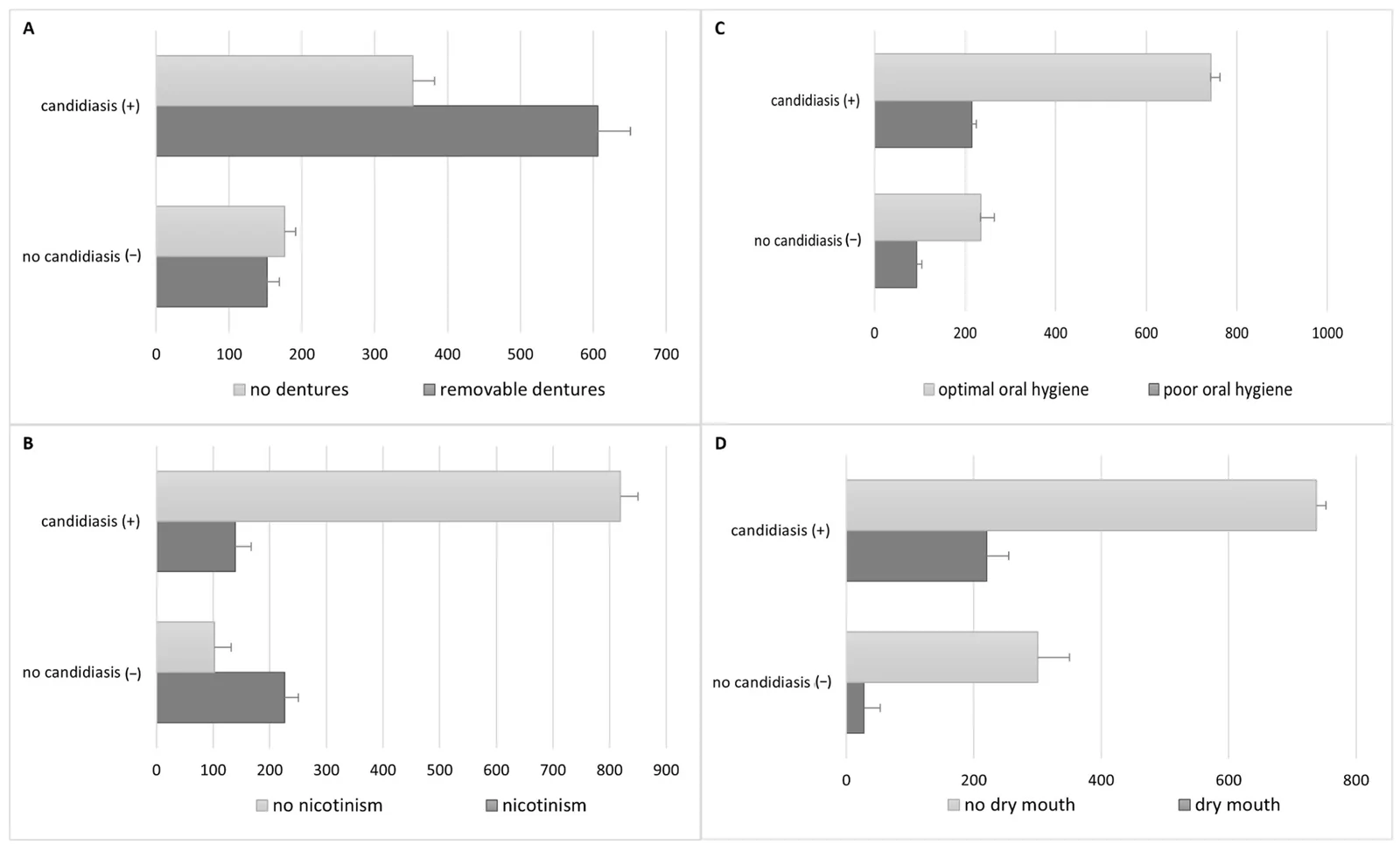
Comparison of infection rates in relation to selected risk factors. (**A**) Use of removable dentures—a statistically significant relationship was found between denture use and the presence of oral candidiasis. (**B**) Nicotinism—no statistically significant relationship with the presence of oral candidiasis. (**C**) Poor oral hygiene—a significant correlation with the occurrence of oral candidiasis. (**D**) Reduced saliva secretion—a statistically significant relationship with the presence of oral candidiasis.

The use of removable dentures was documented among 758 patients (58.94%) (450 female and 308 male subjects), of whom 606 individuals exhibited a favorable mycological examination outcome (Figure 3A).

A total of 170 subjects (122 women and 48 men) were confirmed to have used dentures continuously. The study revealed a statistically significant correlation between the utilization of removable dentures and the prevalence of oral candidiasis (*p* = 0.042). A total of 365 subjects (28.38%) reported nicotine dependence (201 women and 164 men), of whom 139 subjects had a positive mycological examination (Figure 3B).

Characteristics of the smoking group were as follows: 1–10 cigarettes/day: 31.06%; 11–20 cigarettes/day: 52.44%; >20 cigarettes/day: 16%. A majority (65%) of diagnosed patients smoked traditional, factory-made cigarettes. Another relatively common form of tobacco selected by respondents was roll-your-own tobacco (21%), while slim cigarettes (6%) and menthol cigarettes (5%) were smoked by few, with a higher prevalence among women than men. The use of electronic cigarettes (e-cigarettes) was reported by 5% of the surveyed population, with a particularly high prevalence among individuals aged 18–30 (29.5%), compared to traditional cigarettes (26.2%). The mean duration of the subject’s addiction was 22 years. The study found no statistically significant correlation between nicotine addiction and oral fungus (*p* = 0.1446). Poor hygiene (API > 70%) was observed in 308 subjects (23.95%) (180 among women and 128 among men), of whom 215 subjects had a positive mycological examination (Figure 3C). The study demonstrated a statistically significant correlation between poor oral hygiene and the occurrence of oral candidiasis (*p* = 0.0473). Decreased salivary secretion was observed in 248 patients (19.28%), comprising 164 women and 84 men, of whom 220 subjects exhibited a positive mycological examination (Figure 3D). The factor was evaluated through the analysis of data obtained from an oral clinical examination. The study revealed a statistically significant correlation between reduced salivary secretion and the occurrence of oral candidiasis (*p* = 0.0447). The data extracted from the analysis demonstrated the coexistence of systemic diseases and conditions that may increase the risk of oral mucosal candidiasis and exacerbate its course (Table 3) in 468 patients (36.39% of the total number of patients) (289 among women and 179 among men), 380 of whom had a positive mycological examination. The present study demonstrated a statistically significant correlation between systemic diseases coexisting with potential immunodeficiencies and the occurrence of oral candidiasis (*p* = 0.0358).

**Figure 4:**
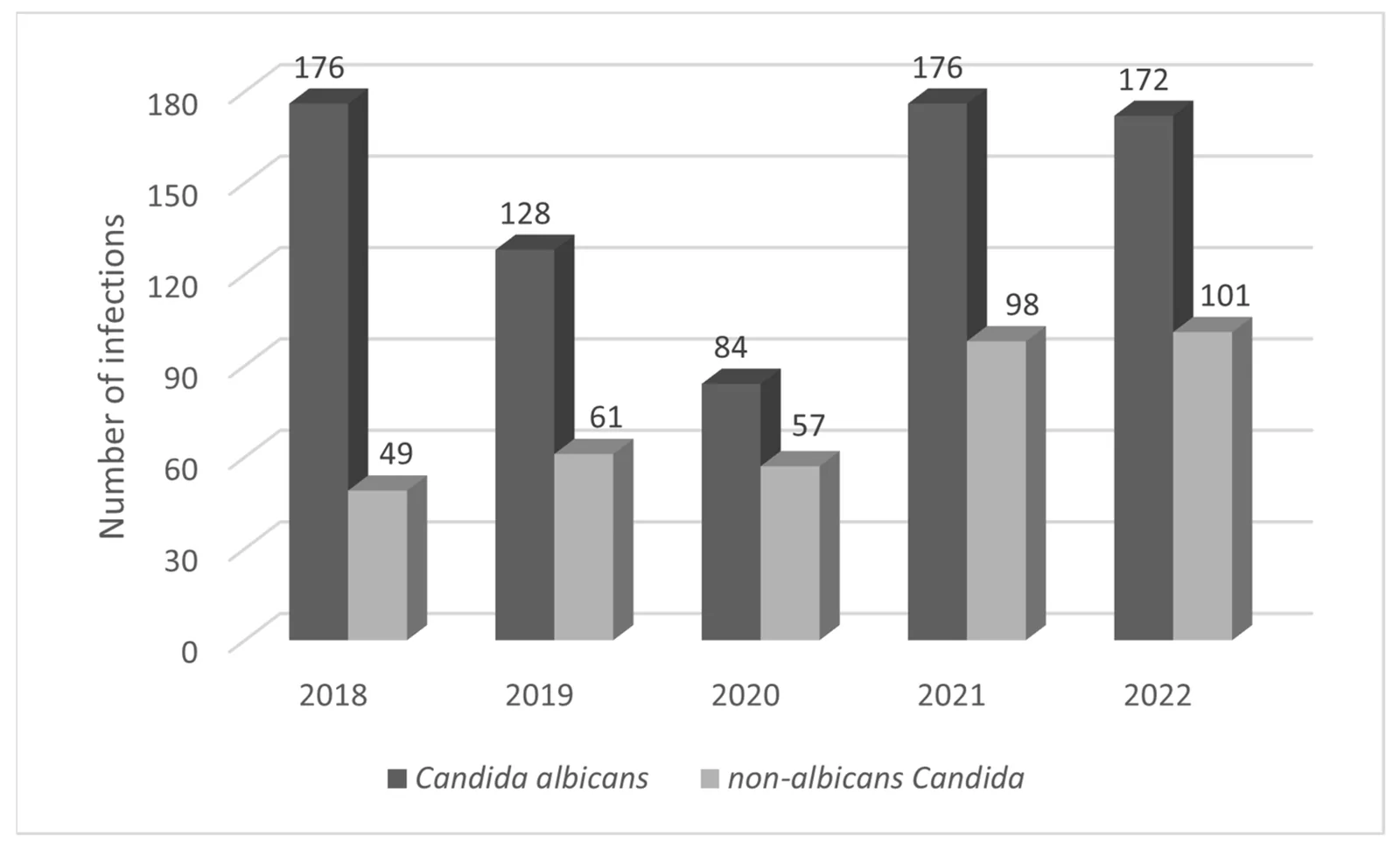
Comparison of the prevalence of infections caused by *C. albicans* and NAC by year (2018–2022).

The most prevalent form of diabetes mellitus was uncompensated, affecting 172 patients (13.37%) with an HbA1c level of ≥6.5% (48 mmol/mol). A statistically significant association was demonstrated between uncontrolled diabetes and the occurrence of oral candidiasis (*p* = 0.0431). The second most common form was systemic glucocorticosteroid therapy/inhaled steroid therapy, affecting 82 patients (6.38%). The most used glucocorticosteroids in this context were prednisone, prednisolone, and methylprednisolone, while betamethasone, budesonide, dexamethasone, and fluticasone were used in inhaled form. The analysis revealed a statistically significant relationship between systemic glucocorticosteroid therapy/inhaled steroid therapy and oral candidiasis (*p* = 0.0272). The use of systemic antibiotic therapy in the six months preceding the study was reported in 59 patients (4.59%) (penicillins, cephalosporins, and macrolides were the most used antibiotics) (the data obtained did not confirm a statistically significant association between systemic antibiotic therapy and oral candidiasis). The incidence of AIDS/HIV was observed in eight patients (0.62%), while other diseases and conditions associated with immunodeficiency (neutropenia) occurred most rarely, in five patients (0.39%). The data analyzed also provided information on the coexistence of other oral mucosal diseases among the 1286 patients. The most common comorbidity was lichen planus (196 patients—15.24%), followed by recurrent oral ulcers (138 patients—10.73%), burning mouth (122 patients—9.49%), oral leukoplakia (118 patients—9.18%), oral mucositis due to radiotherapy/chemotherapy (63 patients—4.90%), geographic tongue (55 patients—4.28%), chronic labyrinthitis (36 patients—2.80%), Sjögren’s syndrome (24 patients—1.87%), lupus erythematosus (9 patients—0.70%), pemphigoid (7 patients—0.54%), pemphigus (6 patients—0.47%), stomatitis associated with anemia (5 patients—0.39%), and graft-versus-host disease (5 patients—0.39%); oral mucosal carcinoma was the least common (4 patients—0.31%). There were 176 *CA* infections in 2018, while there were 128 infections in 2019, 84 infections in 2020, 176 infections in 2021, and 172 infections in 2022. For NAC infections, there were 49 infections in 2018, 61 infections in 2019, 57 infections in 2020, 98 infections in 2021, and 101 infections in 2022 (Figure 4). In 2018–2021, the study found no statistically significant relationship between the distribution of different *Candida* strains (2019 vs. 2020, *p* = 0.1384; 2020 vs. 2021. *p* = 0.0663; 2019 vs. 2021, *p* = 0.4429); such a relationship was noted between 2018 and 2022 (*p* = 0.0290).

The medical records of 106 patients (8.24%) did not contain data on any of the risk factors analyzed in this study, but 56 patients (4.35%) had more than one risk factor for candidiasis.

## 3. Discussion

The primary objective of this retrospective study was to assess the prevalence of various *Candida* strains in patients diagnosed with oral mucosal candidiasis at the Department of Periodontal and Oral Mucosa Diseases (Silesian Medical University in Katowice, Zabrze) from 2018 to 2022. Rising rates of fungal infections are paradoxically linked to medical advances (e.g., cancer treatment, organ transplantation, longer survival of immunocompromised patients), new diagnostic techniques, more invasive procedures, and overuse of broad-spectrum antibiotics [32,33,34,35,36,37,38].

Our study detected a higher proportion of *CA* compared to NAC, consistent with findings from our Center in 2011 and reports from Europe, South America, China, and the Middle East [2,32,33,34,35,36,37]. Epidemiological trends vary by region, diagnostic center, and patient type. At our Center, NAC rose from 22% (2011) to 33.21% in the later period. Bochniak et al.’s 2020 [39] Polish study (228 patients) documented NAC at 26.32%, while Muzaheed et al. in Saudi Arabia (1256 patients) reported 40% [32,33,34,35,36,38,39,40]. In our analysis, 9.8% of strains were *C. glabrata*, in line with previous studies from Europe and North and South America [39,41], though data from India indicate a higher prevalence of *C. tropicalis* [42]. *CA* remains the most common bloodstream infection (BSI) pathogen at 35.9%, but NAC prevalence has risen over two decades, with *C. glabrata* often found in candidemia [43,44]. Compared to 2011, our retrospective analysis identified rare strains (0.82%), including *C. stellatoidea, C. palmioleophila*, and *C. nivariensis*. The incidence of new, often drug-resistant strains (e.g., *C. auris*) underscores the need for precise species identification [10,11,45,46,47]. Multi-strain infections occurred in 8.46% of positive cases, particularly among patients using removable prostheses or affected by immunodeficiencies, aligning with findings by Hertel et al. [2]. During the five-year period, relapses occurred in 18.58% of patients, while Bochniak et al. in Poland reported a 27.63% recurrence [39].

Another objective was to evaluate *Candida* strains’ drug susceptibility and resistance. Compared to our 2011 findings, *CA* showed stable sensitivity to amphotericin B, nystatin, flucytosine, and econazole, but higher sensitivity to ketoconazole, miconazole, and fluconazole. *C. glabrata* maintained similar sensitivity to econazole, with increased sensitivity to nystatin and ketoconazole but decreased sensitivity to amphotericin B, flucytosine, miconazole, and fluconazole [32]. Antifungal resistance is widespread and on the rise, driven by excessive antifungal use (e.g., prolonged therapy, frequent prophylaxis, subtherapeutic doses, and drug sequestration in biofilms) and inadequate infection control [4,19,48,49]. For instance, resistance to azoles surged in the 1990s due to prolonged antifungal therapy in AIDS patients [49]. Mechanisms vary by species and include target enzyme overexpression, drug scavenging pumps, and mutations that diminish drug import [48,50]. Efficacy also depends on drug pharmacokinetics, immune status, and the infection’s site and severity. Because many antifungals (including azoles) rely on the host immune response to combat invasive *Candida*, severe neutropenia may reduce their effectiveness and require additional supportive therapy [49]. The increasing isolation of NAC species poses a significant clinical challenge due to their different resistance profiles: *C. krusei* exhibits intrinsic resistance to fluconazole, *C. lusitaniae* is tolerant to amphotericin B, while *C. glabrata* and *C. parapsilosis* increasingly show reduced susceptibility to azoles and echinocandins, respectively [51,52].

The subsequent objective was to evaluate risk factors for oral candidiasis. In most patients, at least one predisposing factor was identified; only 8.24% had no apparent risk factors, consistent with Bochniak et al. (8%) [39]. Global data also indicate an increasing prevalence of these factors [2,4]. Notably, IL-17 inhibition elevates the likelihood of fungal infections in psoriasis patients, as noted by Campione et al. (2020) and Reich et al. (2021) [53,54]. The most significant risk factor was the use of removable dentures, reported in 47.12% of subjects in our 2011 study, 51.44% in another Polish analysis, and 65.35% in China [32,34,39]. Chronic atrophic candidiasis is the most common multifactorial inflammatory condition in denture users (including partial or complete dentures, removable braces, and obturators), comprising 42.92% of adult cases in our current analysis. Denture wearers generally display a less diverse oral microbiome, which can raise *Candida* carriage [55,56]. Ill-fitting dentures, poor oral hygiene, wearing dentures overnight, and xerostomia further promote *Candida* colonization [57,58,59]. Saliva, which provides both physical cleansing and immune components (e.g., lysozyme, immunoglobulins, glycoproteins, lactoferrin, peroxidase), reduces adhesion and colonization by *Candida*, while diminished salivary flow beneath dentures facilitates fungal adherence and hyphal formation [60,61]. Additionally, mucosal trauma triggers an inflammatory response conducive to *CA* invasion [62]. Management focuses on improving oral and denture hygiene, adjusting or replacing dentures, quitting smoking, avoiding overnight denture use, and administering topical or systemic antifungals. Alternative approaches include microwave disinfection, phytomedicine, antimicrobial photodynamic therapy, and incorporating antifungal agents or nanoparticles into denture resins [63,64,65,66].

Candidiasis frequently affects the oral mucosa, especially in the elderly, newborns, and immunocompromised individuals. In our study, immune disorders were the most significant predisposing factor (*p* = 0.0358) [67]. Uncontrolled diabetes mellitus emerged as the most common systemic factor linked to immune dysfunction (13.37% of subjects), marking an increase from earlier data (2.86%) and from Bochniak et al. (3.28%), but a decrease compared to Yang et al. (6.33%) [32,39,43]. This susceptibility stems from inadequate glycemic control and the direct impact of elevated glucose levels in blood, saliva, and gingival fluid [68,69,70,71]. Systemic or inhaled glucocorticosteroid therapy was statistically significant in 6.38% of participants, a 1.2% increase from earlier data and 0.68% from Bochniak et al., but 7.32% lower than Yang et al. [32,39,43]. In contrast, antibiotic use (4.59% of participants) was not a statistically significant factor, although it was 12.95% lower than Bochniak et al. and 2.21% lower than Hertel et al. [2,32,39]. Antibiotics can disrupt Th17 lymphocyte maturation by eradicating commensal bacteria and can directly affect myeloid cell metabolism, diminishing pathogen clearance [72,73]. Immunosuppressive therapy, a statistically significant factor in 4.2% of participants, increases susceptibility to fungal infections. Candidiasis is the most common opportunistic infection in transplant recipients, comprising 50–60% of cases, prompting a shift toward risk-adapted prophylaxis [2,32,39,74,75]. Radiotherapy (RT) for head and neck cancer was noted in 4.12% of subjects, which was an increase from previous data (2.76%) from Bochniak et al. (1.49%) and Yang et al. (3.64%) [32,39,43]. RT heightens oral toxicity, and nearly 75% of head and neck cancer patients develop fungal infection during treatment [16,76]. HIV infection (0.62% of our patients) leads to pronounced immunosuppression. Oropharyngeal candidiasis (OPC) affects up to 90% of AIDS patients and is primarily due to *CA*, though NAC also appears frequently [2,32,67,77]. Profound or prolonged neutropenia (less than 0.2 × G/L and/or lasting more than nine days) and lymphopenia (especially of CD4+ cells) were observed in 0.39% of our subjects—10.31% lower than in Yang et al. [43]. Such hematological conditions may result from underlying disease or treatments (chemotherapy, RT, immunosuppression) and significantly predispose patients to candidiasis [78].

Cigarette smoke is a significant risk factor for non-communicable diseases and predisposes smokers to infection by impairing immune function (e.g., reduced multinucleated leukocyte activity). In our study, 28.38% of participants were current smokers—comparable to the 29.2% reported by Hertel et al., though higher than the 19.3% reported by Bochniak et al. (no statistical significance) [2,39]. Smoking prevalence was 32.93% among men (higher than 28.38% in women), making it the only risk factor with a statistically significant sex difference. Smokers are seven times more likely to have oral *Candida* infections, partly due to reduced IL-1β, IL-6, and TNF-α responses and nicotine’s enhancement of *CA* biofilm formation and adhesion [68]. Poor oral hygiene (23.95% of subjects) was another statistically significant risk factor. Mun et al. found a positive correlation between active caries and *Candida* isolation [79]. Overall, women were nearly twice as likely as men to present with candidiasis in our study, aligning with findings from our 2011 data and Loster et al., though Yang et al. in China observed a slightly higher male prevalence [34,43,80]. Advanced age also increases susceptibility, often due to reduced immunity and comorbidities; intensive care units see the highest incidence, with invasive procedures contributing to *Candida* pathogenesis [32,34,81]. Our analysis offers insights into *Candida’s* microbiological profiles and antifungal susceptibility, aiding accurate diagnosis and the exploration of alternative therapies when standard treatments fail. Going forward, there will likely be more NAC and rare strains (e.g., *C. auris*), multi-strain infections, and emerging drug resistance. These trends pressure researchers to develop new antifungals and adjunct therapies [9]. Deeper investigations of *Candida* resistance mechanisms and the detection of resistant isolates are increasingly crucial. Because some species are only distinguishable by costly molecular methods, novel and effective diagnostic approaches are urgently needed [10,11,45,46,47].

Given the high azole resistance we observed in NAC species, empiric therapy for uncomplicated oral candidiasis should favor a polyene first line (nystatin or topical amphotericin B), with azoles reserved for isolates proven susceptible or when polyenes are contraindicated; in patients with NAC risk factors such as removable dentures or uncontrolled diabetes, we advise avoiding empiric fluconazole, pairing polyene therapy with denture hygiene and overnight removal, and in recurrent or refractory cases, repeating culture with susceptibility testing to guide targeted therapy rather than escalating azoles empirically.

It is important to note that our work is not without limitations. Primarily, our analysis is based on a single center, and thus future research should be conducted on a larger population across multiple scientific and research centers with a similar periodontal profile to enhance the generalizability of the findings. The analysis of disease entities diagnosed and treated in the clinic is beneficial for monitoring the prevalence of risk factors and changes in their trends, which is crucial for the effective therapy and prevention of the recurrence of the condition [82]. Further limitations include the five-year scope of the review conducted, which encompasses the period of the global pandemic caused by the SARS-CoV-2 virus, during which patient access to specialized healthcare was limited. Additionally, the number of patients included in the review is relatively small, and certain specialized diagnostic tools, such as molecular identification, PCR analysis, and single-methodology assessments of drug resistance, were not available. Furthermore, the study lacks itraconazole and echinocandins MIC data, underscoring the need for additional research on antifungal susceptibility. Moreover, outpatient treatment was conducted instead of inpatient treatment, which may have affected the results due to potential patient nonadherence to prescriptions. Multivariate logistic regression and odds ratios could not be performed due to dataset constraints in this retrospective design, and we have acknowledged these omissions as key limitations of the study.

## 4. Materials and Methods

A retrospective analysis of electronic patient medical records was conducted, focusing on data from medical history and physical examinations that could predispose to candidiasis. This included personal information about examined patients (age, gender), details on candidiasis (the isolated Candida strain(s), whether the condition was primary or secondary, number of recurrences in the last five years, location in the oral cavity such as tongue, palate, cheeks, denture plate, coexisting foci on skin or other mucous membranes, and any coexisting oral bacterial or viral infections), and therapy details (strain sensitivity to antifungal drugs, type and duration of topical or systemic therapy). Concomitant local factors predisposing to candidiasis were also examined, including the use of removable dentures (24/7 or removed overnight, duration of current denture use), tobacco use (number of cigarettes per day, form of tobacco, duration of habit), oral hygiene (API Lange et al. [83]), and saliva secretion (mirror test, resting/stimulated sialometry). Systemic comorbidities with potential immunodeficiencies were assessed, such as diabetes mellitus (hyperglycemia defined by HbA1c), systemic immunosuppressive or glucocorticosteroid therapy (including inhaled), systemic chemotherapy, head and neck radiation therapy, HIV/AIDS, antibiotic therapy in the six months before examination, and other immune deficiency conditions (e.g., neutropenia).

Antifungal susceptibility testing of all Candida isolates was performed according to the European Committee on Antimicrobial Susceptibility Testing (EUCAST) guidelines (versions 8.0–12.0, 2018–2022), using standard broth microdilution methods to determine minimum inhibitory concentrations for amphotericin B, nystatin, flucytosine, econazole, ketoconazole, miconazole, and fluconazole.

### 4.1. The Process of Diagnosing Patients

Based on the patient’s history and clinical exam, candidiasis classification followed Holmstrup et al. [84], identifying three main forms: pseudomembranous (white or cream-colored plaques, sometimes bleeding upon removal), erythematous (red patches that may burn or remain painless under dentures), and hyperplastic (firm, white patches that cannot be wiped off). Other *Candida*-related lesions include prosthetic stomatitis, angular cheilitis, median rhomboid glossitis, and linear gingival erythema. Pre-existing keratinization disorders (e.g., leukoplakia, lichen planus, lupus erythematosus) may also become superinfected by *Candida*.

The clinical diagnosis of oral candidiasis was made based on the quantitative and qualitative results of the mycological examination of the biological material collected in the form of a swab from the oral mucosa and/or dentures.

The material was collected from the patient, and the examination was performed by a diagnostician at an external, certified center (Central Analytical Laboratory, Piastowska 11, 41-800 Zabrze-Polish Centre for Accreditation (PCA) (number AM003)) that has a long-standing collaborative relationship with our department. The mycological study was performed using the culture method—the material was sown on Sabouraud agar with gentamicin and chloramphenicol, Sabouraud Glucose Selective Agar (Thermo Fisher Scientific, Waltham MA, USA). Strain identification was performed by mass spectrometry according to the manufacturer’s instructions for the Vitek MS Prime analyzer (Biomerieux, Craponne, France) using the Vitek MS Prime knowledge base (Biomerieux, Craponne, France). Examination and interpretation of drug susceptibility test results were conducted according to the current EUCAST recommendations: EUCAST version-8.0 (2018), 9.0 (2019), 10.0 (2020), 11.0 (2021), 12.0 (2022).

### 4.2. Statistical Analysis

In this study, we primarily performed descriptive analyses (frequencies, percentages, means, and standard deviations, where appropriate) to characterize the demographic variables, types of *Candida* infections, and distribution of potential risk factors. Since most comparisons involved categorical data (e.g., presence vs. absence of infection, comparison of different *Candida* strains, or evaluation of risk factors), we used the chi-square test of independence (χ^2^). Whenever a 2 × 2 contingency table included cells with low expected frequencies, Yates’ continuity correction was applied to reduce the chance of a Type I error. Given that our outcome measures and predictors were largely nominal or ordinal, no parametric tests (such as the *t*-test) were employed. A *p*-value below 0.05 was considered statistically significant. All computations were carried out using Statistica v. 7.1 PL (StatSoft, Tulsa, OK, USA).

## 5. Conclusions

The study found an increase in oral mucosal infections caused by NAC strains, rare *Candida* strains, or multi-strain infections. Also, a decrease in the efficacy of antimycotics for NAC was observed.

The patients with candidiasis exhibited a prevalence of one or more risk factors (the predominant local risk factor was the use of removable dentures, and the general risk factor was uncontrolled diabetes mellitus). It has been observed that the presence of multiple risk factors in one patient is predisposed to multi-strain infections and more severe clinical symptoms.

The increasing resistance of fungal pathogens to available antifungal medications poses a grave clinical challenge that has the potential to compromise the efficacy of therapeutic interventions, particularly in individuals with compromised immune systems. This phenomenon indicates the need for systematic monitoring of resistance, optimization of therapeutic strategies and intensification of research into new antifungal pharmaceuticals, alternative treatments and adjunctive therapies.

## Figures and Tables

**Table 1:** Quantitative and percentage characteristics of isolated strains in evaluated patients.

Strain	Amount	%
*Candida albicans*	736	66.79
Non-albicans *Candida*	366	33.21
*Candida glabrata*	108	9.80
*Candida tropicalis*	80	7.26
*Candida krusei*	49	4.45
*Candida dubliniensis*	44	3.99
*Candida kefyr*	41	3.72
*Candida lusitaniae*	19	1.72
*Candida parapsilosis*	16	1.45
*Candida* spp. (other rare strains)	9	0.82
Total	1102	100.00

**Table 2:** Characterization of local factors predisposing to oral mucosal candidiasis in examined men and women.

Local Predisposing Factor	Total	Female	Male
Removable prosthesis	758	450	308
Nicotinism	365	201	164
Poor oral hygiene	308	180	128
Dry mouth	248	164	84
24/7 use of removable dentures	170	122	48

**Table 3:** Characteristics of general factors potentially reducing patient immunity predisposing to oral mucosal candidiasis in examined men and women.

General Predisposing Factor	Total	Female	Male
Decompensated diabetes	172	107	65
Systemic glucocorticosteroid therapy/inhaled steroid therapy	82	53	29
Antibiotic therapy in the last 6 months	59	39	20
Systemic immunosuppressive therapy	54	38	16
Radiotherapy to the head and neck area	53	28	25
Systemic chemotherapy	35	18	17
AIDS/HIV	8	4	4
Other diseases and conditions with disease deficits (neutropenia)	5	2	3

## Data Availability

The original contributions presented in this study are included in the article. Further inquiries can be directed to the corresponding authors.
